# The rates of adult neurogenesis and oligodendrogenesis are linked to cell cycle regulation through p27-dependent gene repression of SOX2

**DOI:** 10.1007/s00018-022-04676-6

**Published:** 2023-01-11

**Authors:** Ana Domingo-Muelas, Jose Manuel Morante-Redolat, Verónica Moncho-Amor, Antonio Jordán-Pla, Ana Pérez-Villalba, Pau Carrillo-Barberà, Germán Belenguer, Eva Porlan, Martina Kirstein, Oriol Bachs, Sacri R. Ferrón, Robin Lovell-Badge, Isabel Fariñas

**Affiliations:** 1 Centro de Investigación Biomédica en Red sobre Enfermedades Neurodegenerativas (CIBERNED), Madrid, Spain; 2 Departamento de Biología Celular Biología Funcional y Antropología Física, Universidad de Valencia, 46100 Burjassot, Spain; 3 Instituto de Biotecnología y Biomedicina (BioTecMed), Universidad de Valencia, Valencia, Spain; 4 The Francis Crick Institute, London, NW1 1AT UK; 5 Departamento de Biología Molecular, Universidad Autónoma de Madrid (UAM), Madrid, Spain; 6 Centro de Biología Molecular Severo Ochoa, Consejo Superior de Investigaciones Científicas-Universidad Autónoma de Madrid (CSIC-UAM), Madrid, Spain; 7 Instituto de Investigación Hospital Universitario La Paz (IdiPAZ), Instituto de Salud Carlos III, Madrid, Spain; 8 Department of Biomedical Sciences, University of Barcelona-IDIBAPS, CIBERONC, Barcelona, Spain; 9 Department of Cell and Developmental Biology, Smilow Center for Translational Research, Perelman School of Medicine, University of Pennsylvania, Philadelphia, PA USA; 10 IIS Biodonostia, 48013 Bilbao, Spain; 11 Institute for Research in Biomedicine, 6500 Bellinzona, Switzerland; 12 Max Planck Institute of Molecular Cell Biology and Genetics, 01307 Dresden, Germany

**Keywords:** Adult neural progenitors, Adult neuroblasts, Cyclin-dependent kinase inhibitor, Neural differentiation, ATAC-Seq, RNA-Seq

## Abstract

Cell differentiation involves profound changes in global gene expression that often has to occur in coordination with cell cycle exit. Because cyclin-dependent kinase inhibitor p27 reportedly regulates proliferation of neural progenitor cells in the subependymal neurogenic niche of the adult mouse brain, but can also have effects on gene expression, we decided to molecularly analyze its role in adult neurogenesis and oligodendrogenesis. At the cell level, we show that p27 restricts residual cyclin-dependent kinase activity after mitogen withdrawal to antagonize cycling, but it is not essential for cell cycle exit. By integrating genome-wide gene expression and chromatin accessibility data, we find that p27 is coincidentally necessary to repress many genes involved in the transit from multipotentiality to differentiation, including those coding for neural progenitor transcription factors SOX2, OLIG2 and ASCL1. Our data reveal both a direct association of p27 with regulatory sequences in the three genes and an additional hierarchical relationship where p27 repression of *Sox2* leads to reduced levels of its downstream targets *Olig2* and *Ascl1*. In vivo, p27 is also required for the regulation of the proper level of SOX2 necessary for neuroblasts and oligodendroglial progenitor cells to timely exit cell cycle in a lineage-dependent manner.

**Supplementary Information:**

The online version contains supplementary material available at 10.1007/s00018-022-04676-6.

## Introduction

Mammalian stem and progenitor cells are hierarchically organized to produce appropriate numbers of specialized progeny during development and adult tissue renewal. In the subependymal zone (SEZ) of the adult mouse brain, astrocyte-like neural stem cells (NSCs) generate transit-amplifying neural progenitor cells (NPCs) which rapidly divide 3–4 times before they give rise to neuroblasts. After at least an extra division during their migration to the olfactory bulb (OB), neuroblasts exit the cell cycle and differentiate as interneurons [1, 2]. To a lesser extent, NPCs can also give rise to oligodendroglial progenitor cells (OPCs) that turn into corpus callosum (CC) oligodendrocytes [3], as well as to some striatal and CC astrocytes [4]. The generation of progeny in this germinal niche follows, therefore, an orderly program of progressive cell fate specification.

Cell fate decisions are dictated and sustained by master transcription factors (TFs), chromatin regulators and associated networks, but these programs need to act in concert with cell cycle progression and exit, a coordination that is still poorly understood [5]. Members of the Cip/Kip family of cyclin-dependent kinases (CKIs) have been implicated in cell cycle control in the SEZ lineage. Previous analyses have shown that p21^Cip1^ regulates self-renewal of subependymal NSCs [6–8], whereas p27^kip1^ (encoded by the *Cdkn1b* gene) appears to act as a cell cycle inhibitor of adult NPCs [9, 10]. Interestingly, these Cip–Kip CKIs can act beyond cell cycle control, displaying roles in gene expression. In the SEZ, p21 has been shown to transcriptionally regulate *Bmp2* and *Sox2* gene expression [7, 8]. Although a similar role for p27 has not been investigated, p27 promotes the differentiation of fetal cortical NPCs through stabilization of the TF neurogenin 2 [11] and can repress *Sox2* gene expression in embryonic stem and pituitary cells [12, 13]. A non-canonical role of p27 on the transcriptional regulation of specific genes in adult subependymal NPCs could potentially be a mechanism to timely coordinate cell cycle exit with the implementation of expression programs required to drive differentiation of the different cell lineages [14]. However, a comprehensive analysis of p27 actions on gene expression in a somatic cell system has not been performed.

Here, we have analyzed the gene expression and regulatory landscapes modulated by p27 during the transition from NPC proliferation to differentiation in neuronal and oligodendroglial subependymal lineages. We show that p27 participates in timing cell cycle exit through CDK inhibition, and when absent, residual levels of CDK activity allow the cells to engage in extra cell cycles even in the absence of mitogens. Furthermore, using an assay for transposase-accessible chromatin followed by sequencing (ATAC-seq) [15] together with RNA deep sequencing (RNA-seq), we characterize global expression and chromatin changes that require p27 during differentiation. With this approach, we have uncovered a p27-dependent repression of *Sox2*, *Olig2* and *Ascl1* genes at the onset of differentiation. Our data reveal a direct association of p27 with regulatory sequences in these three genes and an additional hierarchical relationship where p27 repression of the *Sox2* gene leads to reduced levels of SOX2-downstream targets *Olig2* and *Ascl1*. In vivo analyses in p27-deficient mice with normal and reduced levels of SOX2 indicate that p27 regulates NPC cycling rate in a SOX2-independent manner, whereas the repressive action of p27 in *Sox2* expression is required for the regulated cell cycle exit of neuroblasts and OPCs. Thus, p27 determines timely cell cycle exit through the regulation of SOX2 levels in both oligodendrocyte and neuron adult lineages.

## Results

### p27 regulates gene expression programs beyond cell cycle at the onset of differentiation

**Fig. 1:**
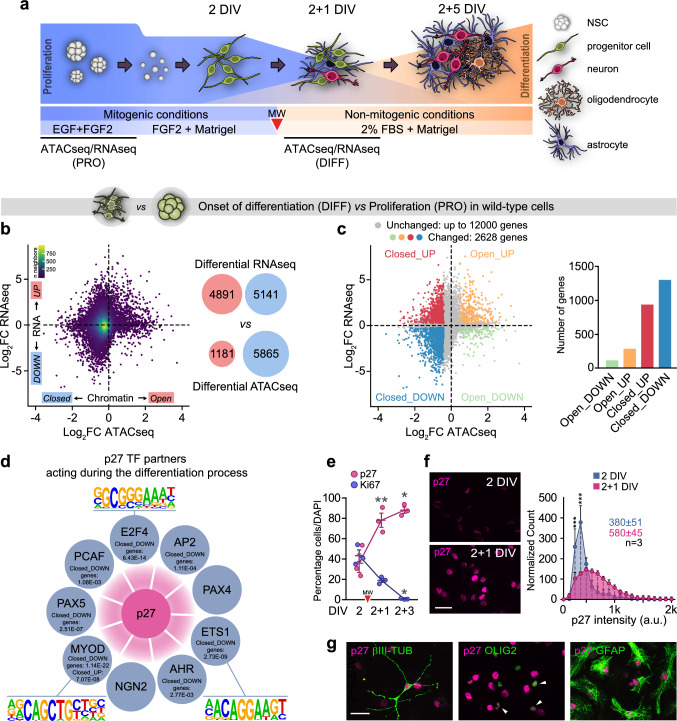
Chromatin and gene expression changes in differentiating NPC cultures. **a** Schematic drawing of the NSC differentiation protocol. A red arrowhead (here and henceforth) indicates mitogen withdrawal to induce differentiation, DIV = days in vitro. **b** Scatterplot of genome-wide chromatin accessibility and mRNA level changes between proliferation and onset of differentiation in WT cells. Open/Closed: genes with promoter-associated differential chromatin accessibility. UP/DOWN genes with differential mRNA expression. Red and blue bubbles indicate the number of genes with sFDR-controlled *p* value < 0.05. **c** Scatterplot from B and summarized histogram colored according to the integrated differential ATAC-seq and RNA-seq defined regulation categories: Closed_UP, Closed_DOWN, Open_UP and Open_DOWN. **d** All known p27 TF partners except PAX4 and NGN2 were identified by LISA as potential regulatory TFs of genes in the Close_UP and/or Closed_DOWN categories during WT differentiation FDR-controlled *p* value and the gene category, along with three DNA sequence motif logos found by HOMER are indicated. **e** Quantification of the percentage of p27^+^ and Ki67^+^ cells during differentiation in WT cultures (p27 *p* value = 0.008, Ki67 *p* value = 0.016, by repeated measures one-way ANOVA). **f** Immunocytochemistry showing expression of p27 (magenta) at 2 and 2 + 1 DIV (left panel). Quantification of the p27 intensity distribution at 2 and 2 + 1 DIV. Median intensity is indicated (right panel). **g** Immunocytochemistry (green) for βIII-TUBULIN^+^ neurons, OLIG2^+^ oligodendrocytes and GFAP^+^ astrocytes and for p27 (magenta) at the end of the differentiation protocol (2 + 5 DIV). *p < 0.05, **p < 0.01, ***p < 0.001. Scale bars: 30 µm

Neurogenesis at the murine SEZ is very active with thousands of neuroblasts born every day [1], but cell cycle exit is not synchronous, making it difficult to assess the temporal resolution of cell-specific global changes. Nevertheless, differentiation can be recapitulated in vitro following mitogen withdrawal (Fig. 1a). Under these conditions, NSC/NPCs stop dividing after 24 h (2 + 1 DIV) and initiate differentiation [16]. Comparative analysis of proliferating 5-day grown neurosphere cells and 2 + 1 DIV cells by ATAC-seq and RNA-seq revealed a great number of differentially accessible (DA) promoters, either closed or open, and differentially expressed (DE) genes, either up- or downregulated, at this “onset of differentiation” (Fig. 1b). The majority of DA promoters in DE genes became closed at 2 + 1 DIV (Fig. 1d), indicating predominant gene repression when cells stop dividing and initiate differentiation. Apart from Closed + DOWN genes, we also found a significant number of Closed + UP genes, likely representing a dynamic transition from accessible to repressed chromatin at cell cycle exit, as chromatin accessibility precedes transcription. We next used the LISA bioinformatic tool [17] for integrative modeling of publicly accessible data on MNase-seq (micrococcal nuclease digestion with deep sequencing, for chromatin accessibility) and ChIP-seq (chromatin immunoprecipitation with deep sequencing, for DNA-associated proteins) in order to identify transcription factors (TF) with significant association to Closed genes. Interestingly, we found several of the strongest regulatory TFs to be known partners of p27 [14] (Table S1). Moreover, motif discovery analysis in Closed + DOWN gene promoters with HOMER [18] also revealed enrichment of consensus binding motifs for reported p27 TF partners, such as E2F4, MYOD or ETS1 (Fig. 1d). Because p27 levels sharply increased at 2 + 1 DIV, when cells stopped proliferation and became Ki67-negative, and remained elevated in differentiated cells (Fig. 1e–g), the data together suggested a putative generalized repressive transcriptional role of p27 at the onset of differentiation.

**Fig. 2:**
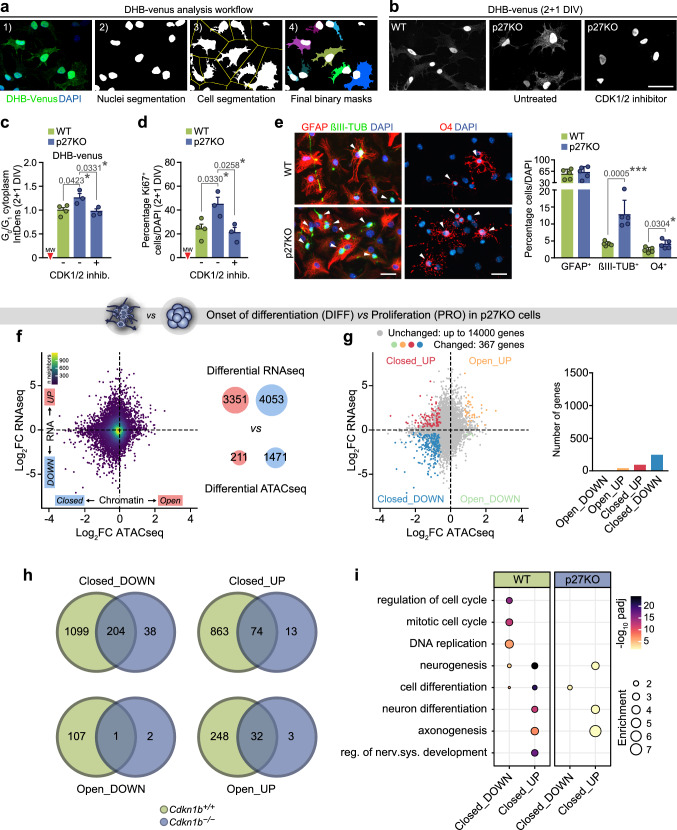
Cell cycling and cell fate decisions in adult NPC cultures are regulated by p27. **a** Main steps during DHB-mVenus bioimage analysis workflow (see “Materials and methods”). **b**, **c** DHB-mVenus signal (white) in WT and p27KO cultures at 2 + 1 DIV after CDK1/2 inhibitor treatment. Comparison of cytosolic gray integrated density (IntDens) of DHB-mVenus fluorescence of cells in G_0_/G_1_ measured by Bioimage Analysis. WT and p27-deficient cultures, either untreated or treated with 1 µM CDK1/2 inhibitor were imaged and scored as fold change relative to WT at 2 + 1 DIV. **d** Percentage of Ki67^+^ cells at 2 + 1 DIV in WT, p27KO and mutant cultures treated with 1 µM CDK1/2 inhibitor. **e** Immunocytochemistry (left) and quantification (right) of βIII-TUBULIN^+^ neurons (green), GFAP^+^ astrocytes (red) and O4^+^ oligodendrocytes at 2 + 5 DIV (left) in WT and p27KO cultures. Arrowheads point out positive cells. DAPI was used to counterstain nuclei. **f**, **g** Scatterplot and histogram as in Fig. 1b, c describing differentiation in p27KO cells. **h** Venn diagrams comparing the number of genes with simultaneous changes in chromatin and expression in wild-type and p27KO cells. **i** Dot plot representation of the functional enrichment analysis of the genes in the overlapping regions shown in h. Graphs represent mean and all error bars show s.e.m. The number of independent biological samples is indicated as dots in the graphs. *p < 0.05; **p < 0.01; ***p < 0.001. Scale bars: 30 µm

*Cdkn1b* mutant mice (p27KO) are larger in size and exhibit hyperplasia in all organs [19–21]. Reported analyses at the single cell level in the developing CNS have indicated that fetal progenitors without p27 undergo one or two extra rounds of cell division before they stop cycling and differentiate, but the underlying mechanism for such a complex cell behavior has not been elucidated [22–24]. In line with the role of p27 in cycling regulation, we detected higher proportions of EdU-incorporating cells at 2 + 1 DIV (mean ± s.e.m.: 9.1 ± 2.0, *n* = 5, vs*.* a wild-type value of 2.2 ± 0.4, *n* = 6, *p* value = 0.0015). Because the decision to reenter the cell cycle is reportedly controlled by the residual level of CDK2 activity at the point of mitotic exit [25, 26], we took advantage of the CDK2 reporter CSII-EF-DHB-mVenus, composed of the C-terminal CDK2 phosphorylation domain of the human DNA helicase B fused to the yellow fluorescent protein mVenus [26]. The reporter is primarily found in the nucleus during mitosis and in newly generated cells, but it translocates to the cytoplasm upon phosphorylation by CDK2 during G1/S transition. Quantitative image analysis of the mVenus cytoplasm/nucleus (C/N) ratio in cells transduced with the reporter (Fig. 2a) indicated that p27-deficient cells that were in G0/G1 (C/N < 0.95) displayed higher cytosolic fluorescence than controls at 2 + 1 DIV, reflecting increased CDK2 activity (Fig. 2b,c). Moreover, cytosolic fluorescence and the proportion of Ki67^+^ cells in p27KO cultures deprived of mitogens could be reduced to wild-type levels by treatment with a pharmacological CDK1/2 inhibitor (Fig. 2b–d). This result indicated that p27-dependent inhibition of CDK2 at cell cycle exit restricts cell cycle progression and suggested that effects of p27 in cell cycle gene expression may be also dependent on CDK activity. Interestingly, although p27-deficient NPCs are overall capable of engaging extra cell divisions, they eventually stop proliferating and fully differentiate by 2 + 5 DIV, but with a bias toward higher proportions of neurons and oligodendrocytes, but not astrocytes (Fig. 2e). This population-specific effect suggested that the role of p27 could go beyond the cell cycle to affect differentiation through specific actions on gene expression, in line with our global analysis.

To test the potential role of p27 in gene expression, we applied the same combinatorial ATAC/RNA-seq approach to cultures obtained from p27KO mice. The number of changes during the differentiation process was remarkably smaller in the absence of p27 resulting in an 85% reduction in the number of genes with changes in either expression or accessibility (Fig. 2f). Intersection of the lists of genes with simultaneous accessibility and mRNA level changes in both genotypes indicated that 88% of the changes in DE genes showing Closed chromatin in the WT process were lost in the absence of p27 (Fig. 2g, h). Gene ontology (GO) analysis identified categories of genes related to “cell cycle control” in the Closed + DOWN group, whereas most genes in the Closed + UP group were related to “neuronal differentiation/neurogenesis” (Fig. 2i). Our analyses suggested that repression of cell cycle-related genes after mitogen withdrawal is subsequently followed by repression of other genes to allow timely differentiation and that repression of both sets of genes is p27-dependent.

### p27 negatively regulates a SOX2-Olig2/Ascl1 axis at cell cycle exit

**Fig. 3:**
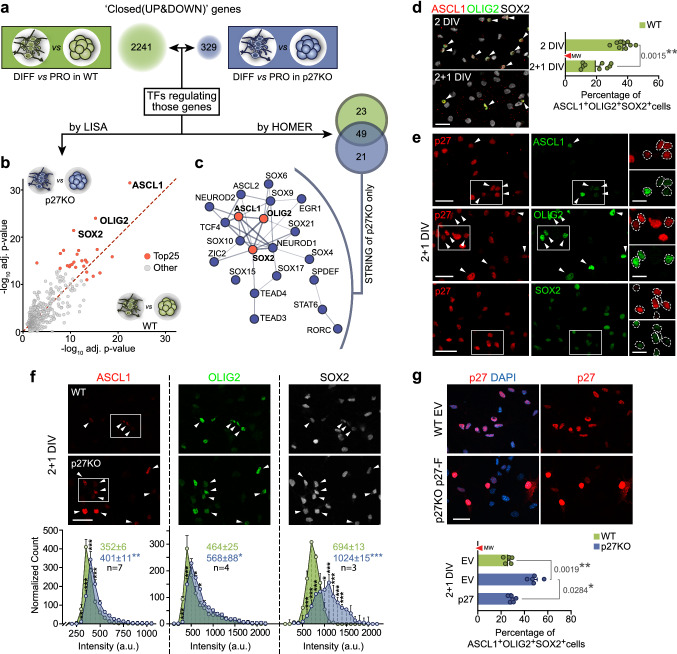
Deregulation of SOX2, ASCL1 and OLIG2 TFs in p27KO cultures at the onset of differentiation. **a** Schematic drawing of the integrative genomic analysis of RNA-seq and ATAC-seq datasets of differentiating vs proliferating wild-type cells (green) and p27KO cells (blue). Numbers in bubbles indicate the amount of UP and DOWN-regulated genes with decreased promoter chromatin accessibility. **b** Comparison of both gene lists with LISA to search for TFs that might be involved in their regulation. The scatterplot shows the statistical significance of TF with each of the gene lists. **c** HOMER search for TF binding motifs in the promoters of genes in both lists. The Venn diagram shows the statistically significant (FDR < 0.05) binding sites found for each comparison. The STRING interaction network shows the 21 TFs with binding sites at the promoters of genes associated with proliferation-to-differentiation transition in p27KO cells. **d** Immunocytochemistry for ASCL1 (red), OLIG2 (green) and SOX2 (white) in WT cultures at 2 and 2 + 1DIV (left) and quantification of the percentage of triple-positive cells in the culture (right). **e** Immunocytochemistry for p27 (red) in combination with ASCL1, OLIG2 or SOX2 (green) in 2 + 1DIV NPCs. **f** Immunocytochemistry for ASCL1 (red), OLIG2 (green) and SOX2 (white) in 2 + 1DIV WT and p27KO NPCs (top panels). Quantification showing the expression distribution of each TF as a result of p27 deficiency. Colored numbers are the median intensity of each genotype (bottom panels). **g** Immunostaining for p27 (red) in WT cultures transfected with an empty vector (EV) and p27KO cultures overexpressing a full-length p27 construct (p27-Flag) (2 + 1 DIV) (top panel). Quantification of the percentage of ASCL1^+^OLIG2^+^SOX2^+^ cells at 2 + 1 in WT and p27KO cultures either transfected with an empty vector (EV) or with a full-length p27 construct. The number of independent biological samples is indicated as dots in the graphs. Graphs represent mean and all error bars show s.e.m. Exact *p* values are indicated in the graphs and legend, being *p < 0.05; **p < 0.01; ***p < 0.001. Scale bars: 30 µm (inserts in **d**: 15 µm)

To address the possibility that p27 was regulating NSC/NPC differentiation through the modulation of a transcriptional landscape, we next sought to identify the network of differentiation-driving transcriptional regulators acting under the control of p27. We analyzed the list of genes with decreased promoter accessibility at differentiation in wild-type and p27KO cells (Fig. 3a) with the LISA tool in order to find TFs already reported in the literature to bind those genes (Fig. 3b) and with the HOMER tool to detect the presence of TF binding sites in their promoters (Fig. 3c). Comparative analysis of the LISA results in wild type *vs* p27KO revealed that the three most relevant regulatory TFs were ASCL1, OLIG2 and SOX2 (Fig. 3b; Table S2). This result was corroborated by HOMER, which found binding sites for 49 TFs likely controlling the differentiation in both genotypes but also for a group of 21 TFs that were more prominently acting when p27 was absent. The protein–protein interaction network of these 21 p27KO-specific TFs placed ASCL1, OLIG2 and SOX2 at the core of the network (Fig. 3c). Taken together, the data suggested that p27 is required at the onset of differentiation to restrict the presence of SOX2, OLIG2 and ASCL1 in a subset of repressed genes.

Identifying SOX2, OLIG2 and ASCL1 as the most prominent TFs potentially involved in the altered differentiation of p27KO cells led us to investigate whether p27 could be acting as a regulator of the corresponding genes. SOX2 is expressed by NSCs and NPCs [27, 28]. ASCL1 is expressed by activated NSCs and by NPCs for both oligodendrocytes and neurons, whereas the presence of OLIG2 in a few NPCs renders them oligodendrogenic [29–31]. Around 40% of all cells in a proliferating culture are SOX2^+^OLIG2^+^ASCL1^+^, but this percentage becomes reduced at 2 + 1 DIV to around 20% (37.3 ± 1.7% at 2 DIV vs 19.5 ± 2.7% at 2 + 1 DIV, *n* = 9, *p* value = 0.0015) (Fig. 3d), in inverse correlation with the increasing levels of p27 (Figs. 1e,f; 3e). In agreement with a repressive action of p27, we scored a significantly higher proportion of cells with increased levels of ASCL1, OLIG2 and SOX2 in differentiating p27KO cells that could be restored to wild-type levels by transduction of a p27 cDNA (Fig. 3f, g).

**Fig. 4:**
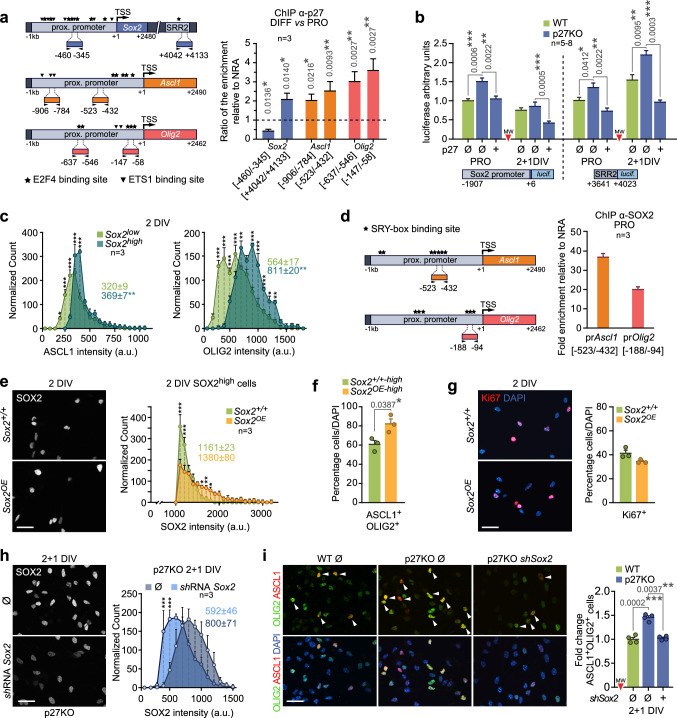
Direct and indirect regulation of Sox2, Ascl1 and Olig2 genes by p27. **a** Schematic drawing of *Sox2*, *Ascl1* and *Olig2* genes and amplicons used in the p27ChIP protocol. *TSS* transcription start site. Stars: putative E2F4 binding sites. Inverted triangles: putative ETS1 binding sites (left panel). Anti-p27 ChIP assay in differentiative (DIFF, 2 + 1 DIV) relative to proliferative (PRO, spheres) conditions. Binding to the *Sox2* promoter and enhancer regions and to the *Ascl1* and *Olig2* proximal promoters is represented as the ratio of enrichment normalized to a non-related antibody (NRA) (right panel). **b** Luciferase reporter assay in PRO and DIFF conditions in WT, p27KO cultures and p27KO cells transfected with a full-length p27 construct. Transcription activity is represented as arbitrary units relative to WT during proliferation. **c** Frequency histograms showing ASCL1 and OLIG2 levels among SOX2^low^ and SOX2^high^ populations at 2 DIV of differentiation. Colored numbers are the median intensity of each population. **d** Schematic drawing of *Ascl1* and *Olig2* genes and amplicons used in the SOX2 ChIP protocol. Stars: putative binding sites for the SOX family (SRY box bs) (left panel). Anti-SOX2 ChIP assay during proliferation. Binding is represented as fold enrichment relative to a non-related antibody (NRA) (right panel). **e** Immunocytochemistry showing SOX2 expression (white) after Sox2 overexpression at 2 DIV (left panel). Histogram showing SOX2 levels in the 30% brightest cells (SOX2^high^) of WT and overexpressing (*Sox2*^*OE*^) cultures at 2 DIV. Colored numbers indicate the median intensity (right panel). **f** Percentage of ASCL1^+^OLIG2^+^ cells among the SOX2^high^ population during 2 DIV of differentiation of WT (*Sox2*^+*/*+*−high*^) and SOX2 overexpressing cultures (*Sox2*^*OE−high*^). **g** Representative images and quantification of the percentage of Ki67^+^ progenitors at 2 DIV after SOX2 overexpression (*p* value = 0.1552). **h** Immunocytochemistry showing SOX2 expression (white) (left panel) and the corresponding quantification (right panel) in p27KO cultures after *Sox2* downregulation by *sh*RNA*Sox2* at 2 + 1 DIV. Colored numbers indicate the median intensity (*p* value = 0.0604). **i** Representative images (left) and quantification (right) of the ASCL1^+^OLIG2^+^ population at 2 + 1 DIV after transfection with a *sh*RNA*Sox2* in p27-deficient cells. An empty vector (Ø) was used as a negative control. Data are represented as fold change relative to WT. Graphs represent mean and all error bars show s.e.m. The number of independent biological samples is indicated as dots in the graphs. Exact *p* values are indicated in the graphs and legend, being *p < 0.05; **p < 0.01; ***p < 0.001. Scale bars: 30 µm

We next set up to evaluate direct physical interaction of p27 with regulatory regions in their coding genes under proliferative and differentiative conditions. Highly conserved regulatory regions in the *Sox2* gene include a core promoter [32] and a number of enhancers organized in Sox2-regulatory regions (SRR), where SRR1 contains enhancers (N) 2 and 3 and SRR2 contains N1, 4 and 5 [33, 34] and is active in the adult SEZ [33]. As for *Olig2* and *Ascl1*, the in silico analysis of the 2 kb sequence upstream of their transcription initiation site combining Genomatix and EPD tools revealed the presence of several putative binding sites for E2F4 and ETS1 TFs, some of them clustered in the proximal promoter (− 1 kb) (Fig. 4a). Therefore, we performed chromatin immunoprecipitation (ChIP) with anti-p27 antibodies in wild-type cultures to detect the binding of p27 to several of these regulatory regions (Sox2: [− 460, − 345], Sox2 SRR2: [+ 4042, + 4133], Olig2: [− 637, − 546] and [− 147, − 58], and Ascl1: [− 906, − 784] and [− 523, − 432]). In proliferative cells we could detect binding of p27 to the *Sox2* proximal promoter and the SRR2 enhancer and to the proximal promoters of *Olig2* and *Ascl1* (ratio p27-IP over NRA control ranging between 1.82 and 3.70). At the onset of differentiation, when p27 levels increased abruptly, the presence of p27 in the SRR2 enhancer as well as to all the tested regulatory regions in *Olig2* and *Ascl1* promoters was strengthened two–threefold (Fig. 4a). The results on the *Sox2* promoter and SRR2 enhancer were confirmed by means of specific luciferase reporters: The activities of Sox2prom[− 1907, + 6]-luc and SRR2[+ 3641/ + 4023]-luc) [35] constructs were higher in proliferating mutant cells, dropped or increased upon differentiation, respectively, and could both be repressed by reintroduction of p27 (Fig. 4b).

Interestingly, the levels of OLIG2 and ASCL1 quantitatively correlated with those of SOX2 at the single cell level (Fig. 4c), suggesting the possibility that p27 could also be exerting its transcriptional repression effect on *Olig2* and *Ascl1* indirectly through its control of *Sox2*. Indeed, when we performed ChIP with anti-SOX2 antibodies, we could detect strong binding to the proximal promoters of both TFs (Olig2 [− 188, − 94] and Ascl1 [− 522, − 432]) (Fig. 4d). Consistently, overexpression of Sox2 by viral delivery of a Cre-recombinase to cultures obtained from R26-loxP-STOP-loxP-Sox2-GFP mice [36] resulted in increased proportions of ASCL1^+^OLIG2^+^ cells without effects on proliferation (Fig. 4e–g). The hierarchical relationship of SOX2 over *Olig2* and *Ascl1* suggested the possibility that the increase in the proportion of ASCL1^+^OLIG2^+^ NPCs resulting from a lack of p27 could be rescued by lowering SOX2 levels. Indeed, reduction in SOX2 protein levels, following nucleofection of a specific *Sox2* shRNA in p27-deficient cultures, rescued the proportion of OLIG2^+^ASCL1^+^ NPCs as efficiently as reintroducing p27 (Fig. 4h, i). Our data together indicated that p27 can potentially act as a repressive regulator of the *Sox2*, *Olig2* and *Ascl1* genes through physical binding but, functionally, its direct repressive action on the *Sox2* gene is sufficient to reduce the downstream expression of *Ascl1* and *Olig2*.

### Regulation of Sox2 by p27 is required for timely cell cycle exit in adult neurogenesis and oligodendrogenesis in a cell context-dependent manner

**Fig. 5:**
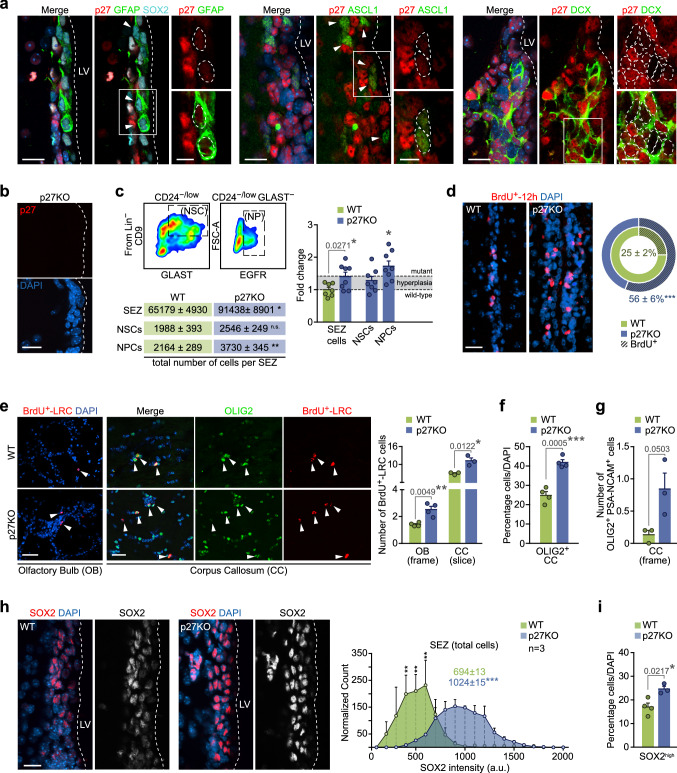
p27 determines the rate of NPC-sustained neurogenesis and oligodendrogenesis in the adult SEZ. **a** Immunohistochemistry showing expression of p27 (red) in GFAP^+^(green)/SOX2^+^ (cyan) NSCs, ASCL1^+^ (green) NPCs and DCX^+^ (green) NBs in the SEZ of WT mice. **b** Immunohistochemistry for p27 (red) in the SEZ of p27KO mice. **c** Representative FACS plots showing GLAST and CD9 staining of Lin^−^ CD24^−/low^ fraction. NSCs are identified by CD9^high^ levels in the GLAST^+^ cells while the Lin^−^ CD24^−/low^ GLAST^−^ region contains EGFR^+^ NPCs (top left panel). Total number of NSCs (*p* value = 0.2400) and NPCs (*p* value = 0.0046) analyzed by FACS in the SEZ as fold change of p27KO relative to WT mice (bottom left panel) (NSCs: n.s., *p* value = 0.5585; NPCs: *p* value = 0.0432). Dashed line represents the observed increment in p27KO number of cells owing to general hyperplasia (right panel). **d** Immunohistochemistry for BrdU (red) within the SEZ of WT and p27KO mice. Percentage of cells that incorporated BrdU in the 12 h prior to euthanasia (BrdU^+^-12 h) in each genotype. **e** Immunohistochemistry showing the staining of BrdU-LRC (red) in the glomerular layer of the olfactory bulb (OB), and of BrdU-LRC (red) and OLIG2 (green) in the *corpus callosum* (CC) of WT and p27KO mice. Arrowheads indicate double-positive cells (left panel). Quantification of the number of BrdU-LRC cells in the OB and CC of wild-type and p27-deficient mice (right panel). **f** Percentage of OLIG2^+^ oligodendrocytes in the CC of WT and p27KO adult brains. **g** Quantification of the number of PSA-NCAM^+^OLIG2^+^ cells per frame in the CC of WT and p27KO mice. **h** Immunohistochemistry showing the expression of SOX2 (red and white) in the SEZ of WT and p27-deficient mice (left panel). Histogram showing quantification of SOX2 intensity. Colored numbers are the median intensity of each population (right panel). **i** Percentage of cells that express high levels of SOX2 in the SEZ of WT and p27-deficient mice. DAPI was used to counterstain nuclei. *LV* lateral ventricle. Graphs represent mean and all error bars show s.e.m. The number of independent biological samples is indicated as dots in the graphs. Exact *p* values are indicated in the graphs and legend, being *p < 0.05; **p < 0.01; ***p < 0.001. Scale bars: **a**, **b**, **h**, 20 µm (inserts 10 µm); **d**, **e**, 30 µm

Next, we decided to test the uncovered regulation of these three TFs by p27 and its functional consequences in the intact SEZ. In postnatal mice, p27 reportedly exhibits a generalized expression in migrating neuroblasts and in young OB neurons with a caudal^low^–rostral^high^ gradient [37], but distribution of the protein in the adult was so far unknown. Analysis with antibodies specific to p27 in young adult (2-month-old) mice indicated that the protein is barely detectable in adult GFAP^+^ NSCs and shows low and variable levels in ASCL1^+^ NPCs, while a strong signal is detected in DCX^+^ cells (Fig. 5a, b), suggesting that p27 levels increase with lineage progression and is maximal in neuroblasts, in line with our in vitro data.

Adult *Cdkn1b* mutant mice have larger bodies [19], and we could also observe enlarged SEZs (volume, in μm^3^ × 10^6^ ± s.e.m.: 21.3 ± 1.8 vs a wild-type value of 15.3 ± 0.9, *n* = 3, *p* value = 0.0411) and OBs (6.0 ± 0.1 vs a wild-type value of 4.7 ± 0.1, *n* = 3, *p* value = 0.0007)*.* Consistent with this, SEZ homogenates showed a 40% increase in cell yield (Fig. 5c); however, when we scored specific cell populations by flow cytometry [38, 39], we found that the increase in NSCs (CD24^−/low^GLAST^+^CD9^high^ cells) was within the range of the generalized hyperplasia of the tissue, whereas NPCs (GLAST^−^CD24^−/low^EGFR^+^ cells) were significantly overrepresented with an increase of 70% in *Cdkn1b* mutant mice (Fig. 5c). In agreement with this, 2-month-old mice intraperitoneally injected with 7 pulses of BrdU, one every 2 h, during the 12 h period preceding euthanasia, evidenced that overall BrdU incorporation rate doubled in p27KO mice (Fig. 5d), whereas GFAP^+^ cells exhibited a normal BrdU-labeling rate ****(percentage of GFAP^+^ that were BrdU^+^  ± s.e.m.: 8.9 ± 2.0 vs. a wild-type value of 10.2 ± 1.6, *n* = 3, *p* value = 0.5980). The data confirmed that p27 is a specific regulator of NPC cycling, as previously reported [9, 10]. Regarding cell progeny output, we found higher proportions of BrdU^+^ label-retaining cells (LRCs) in the OB glomerular layer and in the CC of mice injected with 7 pulses of BrdU four weeks before euthanasia (Fig. 5e). The increase in the density of LRCs in the CC correlated with more OLIG2^+^ cells that were also positive for PSA-NCAM (characteristic of oligodendroglia generated in the SEZ [3]) (Fig. 5f, g). Therefore, p27 limits both neurogenesis and oligodendrogenesis in the adult brain.

**Fig. 6:**
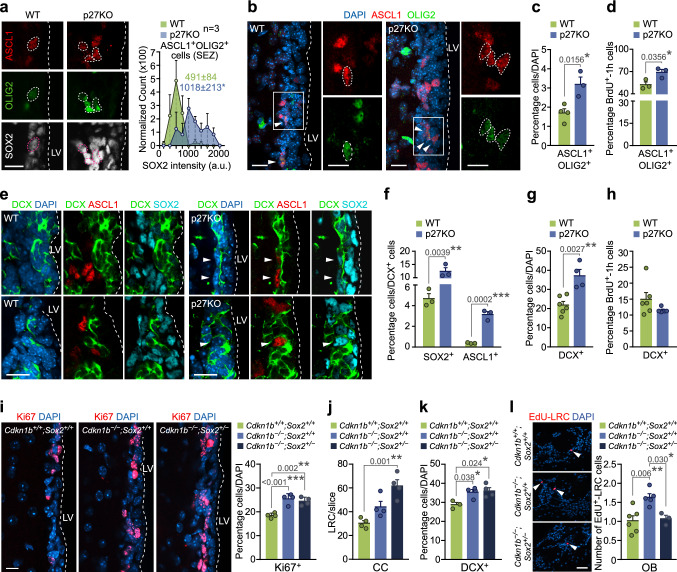
p27-dependent levels of SOX2 regulates timely cell cycle exit in adult neurogenesis and oligodendrogenesis. **a** Representative immunohistochemistry images for ASCL1 (red) and OLIG2 (green) and SOX2 (white) in the SEZ of WT and p27KO mice. Triple-positive cells are delineated (left). Quantification of SOX2 expression intensity in ASCL1^+^OLIG2^+^ cells in the SEZ of WT and p27KO mice (right). **b** Representative immunohistochemistry images for ASCL1 (red) and OLIG2 (green) in the SEZ of WT and p27KO mice. Arrowheads indicate double-positive cells. **c** Percentage of ASCL1^+^OLIG2^+^ cells in the SEZ of adult WT and p27KO mice. **d** Percentage of proliferating cells (BrdU-1h^+^) among the ASCL1^+^OLIG2^+^ population as a result of p27 deficiency. **(e)** Immunohistochemistry showing expression of ASCL1 (red), DCX (green) and SOX2 (cyan) in the SEZ of WT and p27KO mice. Arrowheads indicate triple-positive cells. **f** Quantification of the percentage of DCX^+^ NBs that are positive for SOX2 or ASCL1 in the SEZ of WT and p27KO mice. **g** Percentage of DCX^+^ NBs produced in the adult SEZ. **h** Percentage of proliferating cells (BrdU-1h^+^) among the DCX^+^ p27-mutant population. **i** Immunohistochemistry for Ki67 (red) in the adult SEZ of WT, p27KO (*Cdkn1b*^*−/−*^*;Sox2*^+*/*+^) and p27-deficient, *Sox2* heterozygous (*Cdkn1b*^*−/−*^*;Sox2*^+*/−*^) mice (left panel). Percentage of Ki67^+^ cells (*p* value = 0.0004 by one-way ANOVA) (right panel). **j** Number of EdU-LRC cells in the CC of *Cdkn1b*^+*/*+^*;Sox2*^+*/*+^, *Cdkn1b*^*−/−*^*;Sox2*^+*/*+^ and *Cdkn1b*^*−/−*^*;Sox2*^+*/−*^ mice (*p* value = 0.002 by one-way ANOVA). **k** Percentage of DCX^+^ cells in the SEZ of *Cdkn1b*^+*/*+^*;Sox2*^+*/*+^, *Cdkn1b*^*−/−*^*;Sox2*^+*/*+^ and *Cdkn1b*^*−/−*^*;Sox2*^+*/−*^ mice (*p* value = 0.020 by one-way ANOVA). **l** Immunohistochemistry and quantification of the number of BrdU-LRC (red) reaching the glomerular layer of the OB of *Cdkn1b*^+*/*+^*;Sox2*^+*/*+^, *Cdkn1b*^*−/−*^*;Sox2*^+*/*+^ and *Cdkn1b*^*−/−*^*;Sox2*^+*/−*^ mice (*p* value = 0.005 by one-way ANOVA) DAPI was used to counterstain nuclei. *LV* lateral ventricle. Graphs represent mean and all error bars show s.e.m. The number of independent biological samples is indicated as dots in the graphs. Exact *p* values are indicated in the graphs and legend, being *p < 0.05; **p < 0.01; ***p < 0.001. Scale bars: **a**, **e**, **i** 20 µm (inserts 10 µm); **l** 30 µm

Increased production of neurons and oligodendrocytes in the absence of p27 could be the result of an enlarged population of proliferating NPCs in the SEZ. However, our data indicated that p27 negatively regulates the levels of TFs SOX2, ASCL1 and OLIG2, largely by transcriptional repression of the *Sox2* gene. Analysis of the SOX2 protein by quantitative image analysis in immunostained SEZ sections revealed higher levels *per* cell and increased proportions of SOX2^high^ cells also in vivo (Fig. 5h, i). In contrast to the culture situation, OLIG2 is restricted to a minority (around 2%) of ASCL1^+^SOX2^+^ NPCs that behave as OPCs and specifically generate oligodendrocytes in vivo [3, 29]. Analyses of these OLIG2^+^ASCL1^+^ cells in p27KO mice revealed increased levels of SOX2 per cell (Fig. 6a) and higher proportions of OPCs that, furthermore, proliferated more actively as shown by increased BrdU incorporation rate after a 1-h chase (Fig. 6b–d). As with regard to the neuronal lineage, doublecortin (DCX)^+^ early neuroblasts generated from NPCs can proliferate once or twice as they retain EGFR and are sensitive to mitogens before they exit cell cycle as late nondividing EGFR^−^ neuroblasts that constitute, by far, the largest population in the SEZ neurogenic niche [2, 38]. We found that DCX^+^ neuroblasts in wild-type mice had very low or undetectable levels of SOX2 and ASCL1 [40, 41] together with high levels of p27 (Fig. 5a, b), whereas SOX2 and ASCL1 levels remained abnormally high in p27KO neuroblasts (Fig. 6e, f). Although the absence of p27 also resulted in increased proportions of neuroblasts in the SEZ, it did not alter their proliferation rate, as determined by a 1-h chase after a single injection of BrdU (Fig. 6g, h). The results together indicated that, in vivo: (1) p27 regulates the cycling of NPCs, including those of oligodendrocytes, but not of neuroblasts, and (2) p27 restricts the levels of SOX2 in both OPCs and neuroblasts.

Because of the dual action of p27, we next decided to test whether increased levels of SOX2 played a role in the *p27KO* phenotype by analyzing *p27KO*;*Sox2* heterozygous [42] compound double mutant mice. Overall, heterozygous levels of SOX2 did not reduce the increased proportion of Ki67^+^ proliferating cells resulting from the deletion of p27, indicating that cell cycle regulation in NPCs is SOX2-independent (Fig. 6i). SOX2 effects in neural cells are dosage-sensitive, but also context-dependent [43], and therefore, we decided to analyze oligodendrogenesis and neurogenesis separately. In the postnatal forebrain, SOX2 is needed for the expansion of OPCs during developmental and induced myelination and its levels increase at the onset of differentiation and regulate the production and maturation of myelinating oligodendrocytes [43]. Reduced SOX2 levels in a p27-null background did not restore the increased production of oligodendrocytes; on the contrary, we detected more newly generated oligodendrocytes, as reflected in higher numbers of EdU^+^-LRCs in the CC of the compound mutants injected with the nucleoside four weeks before their analysis (Fig. 6j). Our data indicated that p27 regulates the cycling of OPCs, but the timing of cell cycle exit for differentiation into oligodendrocytes is determined by the level of SOX2.

We next evaluated effects of p27 and SOX2 in neurogenesis. In fetal NPCs, SOX2 typically suppresses premature neurogenesis, likely by counteracting the activity of proneural bHLH TFs [44, 45]. In the adult brain, SOX2 also maintains proliferative NPCs undifferentiated, and if SOX2 levels are reduced below 50%, the NPC pool is depleted, resulting in impaired neurogenesis [41]. In contrast to the oligodendrogenic lineage, SOX2 levels are drastically downregulated at the neuroblast stage, concomitant with a dramatic increase in p27, as shown before. Compound p27KO; *Sox2*^±^ mice still displayed an increased number of DCX^+^ neuroblasts (Fig. 6k), but, interestingly, a dosage reduction in SOX2 levels restored p27KO increased numbers of newly generated OB neurons to WT levels (Fig. 6l). The data reveal a need for p27-dependent repression of stemness TF SOX2 for neuroblasts to timely abandon their last cell cycle before they differentiate into OB neurons. The data together indicate that the regulation of SOX2 dosage by the level of p27 in neuroblasts and OPCs determines cell cycle withdrawal timing and the balance between progenitor cell population expansion and production of specialized cell types.

## Discussion

Arrest of the cell cycle has to be properly coordinated with a switch in gene expression from an undifferentiated to a committed cell-specific profile [5]. We have approached this issue with a combined analysis of the cell state-defining transcriptome and the cell capacity-defining epigenome at the cell transition between mitogen-driven proliferation and mitogen-withdrawal differentiation in the presence and in the absence of the cell cycle inhibitor p27. We provide evidence that increased levels of p27 in response to lack of mitogenic stimulation limit CDK2 activity, therefore restricting cell cycle reentry. Concurrently, p27 repression of *Sox2* expression acts as a timer for cell cycle arrest for differentiation. Our data indicate that p27 acts as a cell cycle break for NPCs and that *Sox2* upregulation in the absence of p27 contributes to the exuberant neurogenic phenotype of *Cdkn1b* null mice by delaying cell cycle exit of committed neuroblasts. A previous report in adult mice with a N-terminal truncated p27 also indicated an increased proportion of proliferating NPCs but reduced olfactory neurogenesis [9], which could be explained by these non-canonical actions of the CKI. During oligodendrogenesis, p27 regulates OPC cycling [23, 46–49] and then increases during differentiation to enhance expression of the myelin basic protein gene [50]. In line with this, we show that precise coordination of p27 and SOX2 levels plays a role in the balance between OPC expansion and oligodendrocyte production. Our results explain how cell cycle exit and the onset of differentiation can be coordinated in the adult SEZ through a mechanism based on a dual p27 action on CDK inhibition and gene expression.

SOX2 belongs to the SOXB1 subfamily, which also includes SOX1 and SOX3 [27, 28]. Although SOX2 is widely known as a pluripotency-sustaining transcription factor in ES cells, it plays a very prominent role in neurogenesis, from specification of the neuroectodermal lineage to maintenance of neural competence [51] in a highly dose-dependent manner [40, 52]. Reduction in SOX2 levels limits self-renewal and multipotentiality and promotes NPC cell cycle exit and premature differentiation. In contrast, overexpression of SOX2 not only inhibits the differentiation of multipotential NPCs into neurons and glial cells during fetal development [44, 45], but can also convert cells of other lineages into NSCs [53]. Reported dosage effects of SOX2 indicate that its levels are finely regulated [7, 41, 54, 55], but its transcriptional and posttranscriptional regulation are still far from understood. Here we show that increased levels of SOX2 as a result of impaired transcriptional regulation by p27 have a clear impact on the neurogenic and oligodendrogenic outputs. Our data sustain the concept that SOX2 effects are remarkably dosage-dependent and indicate that the cell context is also essential to explain SOX2 actions. Some target genes of SOX2 have been identified in NPCs, including *Egfr*, TLX (*Nr2e1*) and *NeuroD1* [56–58]. Here we report that SOX2 can directly bind regulatory regions of the *Ascl1* and *Olig2* genes and that their expression directly correlates with SOX2 levels. All these genes are known to regulate the undifferentiated state of NPCs and/or their activity. In addition, some SOX2 target genes encode secreted proteins with direct paracrine and autocrine effects on NSC/NPCs [59] consistent with the idea that SOX2 is a master regulator of expression programs within these cells. Genome-wide binding profile analysis of SOX2 and SOX3 in ES cells, both naïve and specified to NPCs, and in cells differentiated to neurons has revealed an ordered, sequential binding to enhancers associated with a common set of neural genes needed throughout the neurogenic process [60]. Although SOX2 and SOX3 are thought to act redundantly in many contexts, this latter report suggested that SOX2 acts as a pioneer factor that binds neural enhancers for future activation by SOX3, establishing neural competence. Among the silent genes that are pre-bound by SOX2 in ES cells that are later occupied and activated by SOX3 during neural lineage development are *Ascl1* and *Olig2* [60].

A direct role of SOXB1 proteins in cell cycle progression has never been demonstrated, although actions of these regulators in sustaining the undifferentiated state appear linked to a cycling state [44, 45]. We have been able to evaluate the effect of an increased dose of SOX2, and our data also indicate that SOX2 does not play a direct role in cell cycling, but it has to be silenced in neuroblasts to allow their cell cycle exit for differentiation. It has been shown that E2F3a and E2F3b play antagonistic roles in the balance between activation (E2F3b through recruitment of RNA polymerase) and repression (E2F3a together with Rb pocket protein p107) of the *Sox2* gene to achieve a correct dosage in proliferating NPCs [61]. Modulation of *Sox2* expression by cell cycle-related proteins converts *Sox2* into a key gene involved in coordinating cycling and broad developmental potential.

## Materials and methods

### Animals and in vivo manipulations

*Cdkn1b* mice (p27KO) [19] were obtained from Jackson Laboratory and maintained on a C57BL/6 J background. *Cdkn1b*^*−/−*^*;Sox2*^±^ mice [62], their control littermates (*Cdkn1b*^+*/*+^*;Sox2*^+*/*+^*, Cdkn1b*^*−/−*^*;Sox2*^+*/*+^) and Rosa26R-loxP-STOP-loxP-*Sox2*-GFP mice (for *Sox2* overexpression) [36] were maintained on mixed backgrounds. Animals were genotyped by PCR of DNA extracted from ear punch using specific primers (*wild-type* F: GATGGACGCCAGACAAGC; *wild-type* R: CTCCTGCCATTCGTATCTGC; *Cdkn1b* F: CTTGGGTGGAGAGGCTATTC; *Cdkn1b* R: AGGTGAGATGACAGGAGAT) or done by Transnetyx. Housing and experiments were carried out following protocols approved by the Ethics Committee of the Universidad de Valencia (CEEA: A1403250608615) (Spain) and by the Animal Ethics Committee and the UK Home Office (PPL 70/8560) at the Francis Crick Institute (London, UK).

### Tissue preparation and immunohistochemistry

BrdU administration regimes have been previously detailed [63]. Mice were injected intraperitoneally (i.p.) with 50 mg of BrdU (Sigma, B5002) per kg of body weight every 2 h for 12 consecutive hours (7 injections in total) and euthanized either immediately or 28 days later or with just one injection one hour before euthanasia. EdU (Life Technologies, E10187) was administered i.p. at 30 mg/kg every 12 h for 3 consecutive days (6 injections in total) and mice were euthanized 28 days after the last injection. Mice were deeply anesthetized and transcardially perfused with 100 ml of 4% paraformaldehyde (PFA) in 0.1 M phosphate buffer pH 7.4 (PB). For ASCL1 immunodetection, no more than 40 ml of PFA were infused. Brains were dissected out, vibratome-sectioned at 40 μm and serially collected. For immunohistochemistry, sections were washed in PBS and blocked at room temperature (RT) for 1 h in PBS (0.9% NaCl in PB) with 0.2% Triton X-100 supplemented with 10% FBS and then incubated overnight at 4 °C with primary antibodies (1:100 mouse anti-ASCL1, BD 556604; 1:800 rat anti-BrdU, Abcam ab6326; 1:400 chicken anti-DCX, Abcam ab153668; 1:300 goat anti-DCX, Santa Cruz sc-8066; 1:800 chicken anti-GFAP, Millipore AB5541; 1:300 rabbit anti-Ki67, Abcam ab15580; 1:500 rabbit anti-OLIG2, Millipore AB9610; 1:200 rabbit anti-p27, Cell Signaling 3686; 1:700 mouse anti-PSA-NCAM, Millipore MAB5324; 1:600 goat anti-SOX2, R&D AF2018). For BrdU detection, sections were pre-incubated in 2 N HCl for 20 min at 37 °C and neutralized in 0.1 M sodium borate (pH 8.5). Detections were performed with 1:800 fluorescent secondary antibodies (AF488 donkey anti-chicken, Jackson ImmunoResearch 703-545-155; AF488 donkey anti-rabbit, Jackson ImmunoResearch 711-547-003; AF647 donkey anti-goat, Molecular Probes A21447; Cy3 donkey anti-chicken, Jackson ImmunoResearch 703-165-155; Cy3 donkey anti-mouse, Jackson ImmunoResearch 715-165-151; Cy3 donkey anti-rabbit, Jackson ImmunoResearch 711-165-152; Cy3 donkey anti-rat, Jackson ImmunoResearch 712-165-153). EdU detection was carried out using the Click-iT™ Plus EdU Alexa Fluor™ 555 Imaging Kit (ThermoFisher, C10638). Nuclei were counterstained with 1 µg/ml of DAPI and sections were mounted with Fluorsave (Calbiochem). Immunostained series containing 5 coronal sections of the anterior horn of the lateral ventricles (Bregma anteroposterior AP coordinates + 1.10, + 0.74, + 0.38, + 0.02 and − 0.34 mm) were imaged in an Olympus FV10i confocal microscope (Olympus) for SEZ and CC analyses. Cell populations were manually counted using the Olympus and ImageJ/Fiji software, and data were obtained as a percentage of positive cells relative to a subpopulation or to total DAPI labeled cells in the lateral ventricle wall. Between 5 and 10 coronal sections in the medial OB (Bregma AP coordinates + 5 to + 4 mm) were analyzed in neurogenesis studies.

### Neurosphere cultures, differentiation and immunocytochemistry

Methods for SEZ-derived neurosphere NSC cultures and assessment have been previously described in detail [16, 68]. For proliferation assays, NSCs were grown in neurosphere control medium containing 20 ng/ml epidermal growth factor (EGF) (Invitrogen, 53003-018) and 10 ng/ml fibroblast growth factor (FGF) (Sigma, F0291). Importantly, for bulk differentiation assays on secondary neurospheres, 40,000 cells/cm^2^ were seeded for 2 days (2 DIV) in Matrigel®-coated coverslips and in control medium with FGF. Then, mitogens were removed and cells were grown in control medium supplemented with 2% FBS. Differentiating cultures were analyzed 24 h (2 + 1 DIV) or 5 days (2 + 5 DIV) after mitogen withdrawal (see Fig. 1a). For immunocytochemistry, cultures were fixed with 2% PFA in PB for 15 min, blocked (PBS with 0.1% Triton X-100 and 10% FBS) and incubated with primary (overnight at 4 °C) and secondary (1 h at room temperature) antibodies (1:100 mouse anti-ASCL1, BD 556604; 1:100 mouse anti-ASCL1, Guillemot lab; 1:800 rat anti-BrdU, Abcam ab6326; 1:800 chicken anti-GFAP, Millipore AB5541; 1:300 rabbit anti-Ki67, Abcam ab15580; 1:300 mouse anti-O4, Hybridoma Bank rip; 1:500 rabbit anti-OLIG2, Millipore AB9610; 1:200 rabbit anti-p27, Cell Signaling 3686; 1:200 mouse anti-p27, Cell Signaling 3698; 1:600 goat anti-SOX2, R&D AF2018; 1:300 rabbit anti-ßIII-TUBULIN, Sigma T2200; 1:800 AF488 donkey anti-mouse, Molecular Probes A21202; 1:800 AF488 donkey anti-rabbit, Jackson ImmunoResearch 711-547-003; 1:800 AF647 donkey anti-goat, Molecular Probes A21447; 1:1000 biotin horse anti-mouse, Vector Laboratories BA-2000; 1:2000 Cy3-streptavidin, Jackson ImmunoResearch 016-160-084; 1:800 Cy3 donkey anti-chicken, Jackson ImmunoResearch 703-165-155; 1:800 Cy3 donkey anti-mouse, Jackson ImmunoResearch 715-165-151; 1:800 Cy3 donkey anti-rabbit, Jackson ImmunoResearch 711-165-152; 1:800 Cy3 donkey anti-rat, Jackson ImmunoResearch 712-165-153). DAPI (1 µg/ml) was used to counterstain nuclei. 10 µM EdU was administered for 1 h prior to fixation and detected using the Click-iT™ Plus EdU Alexa Fluor™ 555 Imaging Kit (ThermoFisher Scientific, C10638) following the manufacturer’s instructions. For CDK2 inhibition, 2 DIV differentiated cells were treated with 1 µM CDK1/2 inhibitor III (Millipore, 217714) in control medium supplemented with 2% FBS for 24 h. An appropriate dilution of DMSO was used as vehicle.

### Flow cytometry

Characterization of NSC and NPC populations in the adult SEZ was performed as previously described [38, 39]. Roughly, SEZ tissue was dissected, minced and enzymatically digested using the neural tissue dissociation kit (T) (Miltenyi, 130–093-231) in a gentleMACS Octo Dissociator with heaters (Miltenyi). After trypsin inhibition, digested pieces were mechanically dissociated, the cell suspension was filtered through a 40 μm and treated with the Dead Cell Removal Kit (Miltenyi, cat no. 130-090-101) following the instructions of the manufacturer. Finally, the eluted living fraction was pelleted (300×*g*, 10 min) and incubated with the specific cocktail of primary antibodies [38, 39] (1:300 CD24-PerCP-Cy5.5, BD 562360; 1:100 CD31-BUV395, BD 740239; 1:200 CD45-BUV395, BD 565967; 1:20 CD9-Vio770, Miltenyi 130-102-384; 1:20 GLAST-PE, Miltenyi 130-095-821; 1:30 O4-Biotin, Miltenyi 130-095-895; 1:200 Ter119-BUV395, BD 563827; 1:300 AF488 EGF complex, Molecular Probes E13345) and reagents (DAPI, 50 µg/ml) at 4 °C for 30 min. Labeled samples were analyzed using a LSR-Fortessa cytometer (Becton Dickinson) with 350, 488, 561 and 640 nm lasers.

### Chromatin immunoprecipitation (ChIP)

ChIP was performed as previously described [64]. WT neurospheres (3 DIV) and 2 + 1 DIV differentiated cells were cross-linked and chromatin isolated. Chromatin was sheared to an average size of 200–500 bp using a Bioruptor sonicator (Diagenode, UCD-200) and then pre-cleared with 10 µl of protein G magnetic beads (Dynabeads^®^, 10003D) for 3 h at 4 °C with rotation. 5% of pre-cleared chromatin was reserved as input. 5–10 µg of antibody (rabbit anti-p27, Santa Cruz sc-528; goat anti-SOX2, R&D AF2018) or non-related antibody (NRA: Neu (C-18) (rabbit anti-NEU, Santa Cruz sc-284) and Rock2 (N-19) (goat anti-ROCK2, Santa Cruz sc-1852) for p27 and SOX2 immunoprecipitations, respectively) were added and incubated overnight at 4 °C on a rotation wheel. Bound chromatin was retrieved with 20 µl protein G beads for 3 h at 4 °C, washed and submitted to cross-link reversal and protein digestion. Finally, DNA was purified using MinElute PCR purification kit (Qiagen, 28004) following manufacturer’s instructions. ChIP enriched DNA was analyzed by real-time PCR using SYBR green primers (prAscl1[− 906/− 784]-F: TAACCCTGAGTGCCTTCCTG; prAscl1[− 906/− 784]-R: GGAGTCATGAACAGGATAGGTTG;; prAscl1[− 523/− 432]-F: CTGCGGAGAGAAGAAAGGGG; prAscl1[− 523/− 432]-R: TCAGGGAAGGGTTTAGGCAG; prOlig2[− 637/− 546]-F: CTGCAGCAACTGCCACTAAG;; prOlig2[− 637/− 546]-R: CTGTGACCATTTGTGGTTGC; prOlig2[− 147/− 58]-F: TTCATTGAGCGGAATTAGCC; prOlig2[− 147/− 58]-: CGGGAACAATGTGCTTTTC; prSox2[− 460/− 345]-F: ATGAGCGCAGAAACAATGGCA; prSox2[− 460/− 345]-R: ACATAAGGGTGGATGGGGCG; SRR2[+ 4042/ + 4133]-F: AAGAATTTCCCGGGCTCG; SRR2[+ 4042/ + 4133]-R: CCTATGTGTGAGCAAGAACTGTCG;). The reaction conditions were optimized for each primer pair in order to get a single-peak melting curve. The IP and its NRA counterpart were amplified at several dilutions and only considered positive results (Ct of IP < Ct of NRA) if observed in at least two different dilutions. The comparative Ct method was used to calculate fold enrichment levels relative to NRA after normalizing each one to their input.

### Transfection and viral infection of NSCs

For restoration of p27 and SOX2 levels in *Cdkn1b*^−/−^ cultures, NSCs were transfected using the Amaxa NSC Nucleofector Kit (Lonza, VPG-1004) as previously described [16] with either 7.5 μg of pcDNA3.1 and pcDNA3.1-p27-Flag (p27 full-length construct) or 7.5 μg of empty pMSCVpuro (Clontech, 634401) and pSUPER.retro mSox2.3 (*sh*RNA*Sox2* target sequence: 5ʹ-CGAGATAAACATGGCAATCAA-3ʹ), respectively. *Cdkn1b*^+*/*+^ cultures were used as control. Nucleofected cells were plated on differentiative conditions and processed for immunocytochemistry at 2 and 2 + 1 DIV. For overexpression of *Sox2*, Rosa26R-loxP-STOP-loxP-*Sox2*-GFP NSC cultures were infected with Ad-GFP and Ad-CMV-iCre (Vector Biolabs) following the protocol described in [65]. Shortly, 30,000 individual NSCs were infected with a multiplicity of infection (MOI) of 500 in complete medium. After 24 h, viruses were washed, and cells were plated in fresh medium and grown for 5 more days. After passage, Cre-infected (*Sox2*^*OE*^) and control GFP-infected (*Sox2*^+/+^) cultures were plated on differentiative conditions and analyzed at 2 and 2 + 1 DIV. The 30% brightest cells in both conditions were considered as SOX2^high^. In order to study CDK2 activity, *Cdkn1b*^+*/*+^ and *Cdkn1b*^−/−^ cultures were infected with the lentiviral particles carrying the CSII-EF-DHB-mVenus construct [26] as previously described [66]. Infected neurospheres were cultured for 5 days in fresh complete medium, split and differentiated according to the experimental procedure.

### Cloning and luciferase assay

Murine *Sox2* proximal promoter (positions − 1907 to + 6 relative to the transcription initiation site) [32] was retrieved by PCR from C57B6/J genomic DNA with specific primers containing restriction-site target sequences (*prSox2*[− 1907/ + 6]-luc-F: AAAAAACTCGAGAAACTTAAGGAGAACCTGGGG; prSox2[− 1907/ + 6]-luc-R: CCACCAAAGCTTAACAAGTTAATAGACAACCATCCA) and cloned into a pGL3-Enhancer vector (Promega). The correct sequence was checked by Sanger sequencing. *Cdkn1b*^+/+^ and *Cdkn1b*^−/−^ cells were nucleofected (see “Transfection and viral infection of NSCs”) with 7.5 μg pCDNA3.1 or pCDNA3.1-p27-Flag, 0.5 μg of pMAX-GFP and 2 μg of the corresponding reporter construct (*prSox2*[− 1907/ + 6]-luc; *SRR2*[+ 3641/ + 4023]-luc, kindly provided by Dr. Okuda [35], driving the expression of the firefly luciferase and a *Renilla* luciferase plasmid in a 1:20 ratio. After electroporation, NSCs were plated on either proliferative or differentiative conditions. Cells were lysed after 72 h (2 + 1 DIV for differentiations) using the Dual Luciferase Reporter kit (Promega, E1960) and luciferase activity was measured using a Victor3 (Perkin Elmer) reader. Ratio of firefly to *Renilla* luciferase was calculated and represented as arbitrary units (a.u.).

### Imaging and bioimage analysis

Images were acquired with an Olympus FV10i confocal microscope (Olympus). For in vitro experiments, laser settings were first established on WT/control samples at the first time point (e.g., 2 DIV) and kept throughout the whole experiment. Random fields were imaged at the focal plane showing both the highest number of cells on focus and the most intense signal. 2 + 5 DIV differentiation samples were imaged using a Nikon ECLIPSE Ni-U microscope (Nikon) with a Zyla 4.2 sCMOS camera (Andor). Bioimage analysis was performed using the Fiji open-source software package [67]. ImageJ Macro Language scripts were developed to perform unbiased automatic analysis of the in vitro experiments: (1) nuclear signal of EdU, Ki67, SOX2, OLIG2 and ASLC1) was quantified with our previously developed “Cell proliferation HCS” tool [68]. A minimum of 500 cells per culture were scored in all conditions; (2) CDK2 activity was analyzed by a custom workflow (see Fig. 2a; full script available at https://github.com/paucabar/DHB-Venus) that obtains a binary mask for each cell in both DAPI and CSII-EF-DHB-mVenus images. After that, nuclear particles of non-infected cells (mVenus^−^) are discarded and touching or clumped cells are separated by Voronoi partition. Only cytoplasm mVenus-positive (mVenus^+^) mask is obtained after operating (XOR) with the whole cell and nuclear masks. Finally, nuclear (N) and cytoplasmic (C) binary masks are redirected to the original grayscale image to retrieve the mVenus signal. Cells with no mVenus C mask or with a C/N ratio (mean gray value) lower than 0.65 were classified as G0/G1 cells. Residual CDK2 activity was scored as the integrated intensity of the mVenus^+^ C masks of cells in G0/G1. A minimum of 250 cells per culture were scored in all conditions. For in vivo experiments, laser settings were first established on WT tissue and similar regions of interest (ROI) were acquired in an Olympus FV10i confocal microscope. Maximal projection images were generated and the mean gray intensities of nuclear markers (ASCL1, OLIG2, SOX2) were measured with Fiji software. Intensities were represented as frequency histograms normalized to the maximum count in each comparison.

### RNA- and ATAC-seq

3 DIV proliferating neurospheres and NPs at 2 + 1 DIV were harvested for RNA and chromatin extraction. For ATAC sequencing 50,000 cells were centrifuged (500×*g*, 5 min, 4 °C), washed with ice-cold PBS and lysed in cold lysis buffer (10 mM Tris–HCl pH 7.4, 10 mM NaCl, 3 mM MgCl2, 0.1% IGEPAL CA-630). Nuclei were pelleted (500×*g*, 10 min, 4 °C) and tagmented with the Nextera DNA Library Prep Kit (Illumina, FC-121-1030) for 1 h at 37 °C. After transposition, DNA was purified with the MinElute PCR purification kit (Qiagen, 28004) and amplified and barcoded with the NEBNext high-fidelity 2 × PCR master mix (New England Labs, M0541) following manufacturer’s instructions. The amplified library was then purified with XP AMPure Beads (Beckman Coulter, A63880). Library fragments were analyzed and quantified with a 2100 Bioanalyzer (Agilent Technologies). Libraries were sequenced on an Hiseq4000 (Illumina) to achieve an average of 25 million reads per sample in the Advanced Sequencing Facility (ASF) at the Francis Crick Institute. For bulk RNA sequencing, samples were lysed with the QIAzol Lysis Reagent (Qiagen, 79306) and RNA was extracted with the miRNeasy micro kit (Qiagen, 217084) according to the manufacturer’s protocol. RNA quality was assessed using the Agilent RNA 6000 Pico Kit (Agilent Technologies). cDNA was generated using Ovation RNA-seq System V2 (Tecan, 7102-A01), libraries were constructed using Ovation Ultralow System V2 (Tecan, 0344NB-A01) according to the manufacturer’s instructions. Libraries were quantified using the TapeStation (Agilent), pooled in equimolar proportions and sequenced on an Hiseq4000 (Illumina) to achieve an average of 25 million reads per sample.

### Bioinformatic analyses

#### ATAC sequencing

Paired-end sequencing files were analyzed with FastQC, summarized with MultiQC and visually inspected for major quality issues. We used Cutadapt to clip the Nextera 3ʹ R1 and R2 adapters and then Trimmomatic to trim low-quality portions and filter reads shorter than 20 bases. Then, a second round of quality control with FastQC and MultiQC was performed to make sure the outputs were in line with the expected results. High-quality reads were then mapped to the mouse genome with Bowtie2, using the *Mus musculus* GRCm38 (mm10) genome as reference and allowing mate dovetailing. Duplicates were marked on BAM files with MarkDuplicates from Picard tools, insert sizes were calculated with the CollectInsertSizeMetrics tool from SAMtools, and then, peak calling was performed with MACS2, choosing narrow peaks, no model and 0.05 FDR as options. The resulting narrow-peak BED files were manually loaded into the IGV genome browser alongside their corresponding ATAC-seq coverage tracks generated with the bamCoverage function from DeepTools for visual inspection. Biological replicates of each sample were merged and used to generate average metagene plots and per-gene accessibility heatmaps around transcription start site (TSS) genomic coordinates of mouse protein-coding genes with ngsplot. MACS2 narrow-peak files and the corresponding BAM alignment files they were derived from were used as input for differential peak calling with DiffBind. An FDR threshold of 0.05 was chosen for peak differential detection between comparisons. Peaks that were significantly more or less concentrated in one sample compared to its control were considered open and closed, respectively. In order to associate both differential (open and closed) and non-differential peaks to their genomic contexts, we used ChIPseeker with the Gencode M18 mouse GTF annotation. Upstream and downstream regions of 3 Kb were allowed, as well as a flanking gene distance option of 5 Kb. We focused on peaks associated with 5ʹ and promoter regions, as determined by the ChIPseeker annotation, for downstream analysis. In the cases where there was more than one promoter peak associated with the same gene, we kept either the one that was closest to the TSS or the largest overlapping one. Differential promoter peak coordinates were used as input for the DNA motif analysis tool HOMER, with the findMotifsGenome.pl Perl script and the size given option, which instructs the algorithm to look for motifs in all the peak, not just in the region around the center of the peak.

#### RNA sequencing

Raw sequencing files were aligned to the Ensembl mouse transcriptome using STAR, and then, RSEM was used to count raw reads per gene. Genes with less than 1 count per million (CPM) in at least 4 of the samples were discarded. Genomic coverage tracks were generated from BAM alignment files with the bamCoverage function from DeepTools, choosing RPKM normalization. Coverage tracks were loaded into the genome browser IGV for visual inspection. Differential expression analysis between samples was done with DESeq2, and an FDR threshold of 0.05 was used to discriminate between up- or downregulated genes. Similarly to ATAC-seq samples, the biological replicates of each RNA-seq sample were merged and used as input for ngsplot to generate average metagene plots and per-gene mRNA amount heatmaps around transcription start site (TSS) genomic coordinates of protein-coding genes.

#### Integration between ATAC-seq and RNA-seq

The tabular outputs of DiffBind (ATAC-seq) and DESeq2 (RNA-seq) were loaded in R for further processing. We combined both matrices and labeled every gene with its corresponding differential accessibility (DA) and differential expression (DE) analysis results, i.e., DA *Closed*, *Open* or *Unchanged*, and DE *UP*, *DOWN* or *Unchanged)*. For downstream functional analysis, we focused on only genes with significant differences in both modalities simultaneously: *Closed_UP*, *Closed_DOWN*, *Open_UP* and *Open_DOWN*. Functional enrichment analysis of differential genes was carried out using STRING, Mousemine, GOrilla, REVIGO, Panther, Enrichr, Appyter and the ClusterProfiler R package. The AnimalTF and UNIPROT databases were used to extract information about mouse TFs and TF families. The Epigenetic Landscape In Silico deletion Analysis (LISA) tool [17] was used to discover associations between our target gene lists and regulatory TFs and chromatin factors based on the Cistrome DNase and ChIP-seq database (CistromeDB). Density scatterplots, Venn diagrams and violin plots were generated in R with custom code.

### Statistical analysis

All statistical tests were performed using the GraphPad Prism Software (v9.4). Data were analyzed for normality using the Shapiro–Wilk normality test. Analyses of significant differences between means for variables displaying normal distribution were carried out using the unpaired or paired two-tailed Student *t* test for two variables or one-way ANOVA with Tukey post hoc test for more than two groups. Similarly, variables that did not follow a normal distribution were analyzed with Mann–Whitney *U* test or Kruskal–Wallis test, when appropriate. Two-way ANOVA was used in frequency distribution analyses. Relative values (normalized values and percentages) were first normalized by using a log or arcsin transformation, respectively. Data are presented as the mean ± standard error of the mean (s.e.m) unless otherwise stated. The number of experiments carried out with independent cultures/animals (*n*) is either shown as dots in the graphs or listed in the Figure Legends. Statistical significance in the violin plot was assessed by the Wilcoxon signed-rank test as implemented in the ggpubr R package (*P < 0.05, **P < 0.01, ***P < 0.001 and ****P < 0.0001).

### Supplementary Information

Below is the link to the electronic supplementary material.

Supplementary file1 (XLSX 42 KB)

## Data Availability

RNA-seq and ATAC-seq datasets have been deposited in GEO under accession number GEO: GSE196329.
